# The effect of Fenugreek seed dry extract supplement on glycemic indices, lipid profile, and prooxidant-antioxidant balance in patients with type 2 diabetes: A double-blind randomized clinical trial

**DOI:** 10.34172/jcvtr.33231

**Published:** 2024-09-20

**Authors:** Fatemeh Chehregosha, Leila Maghsoumi-Norouzabad, Majid Mobasseri, Laleh Fakhr, Ali Tarighat-Esfanjani

**Affiliations:** ^1^Student Research Committee, Tabriz University of Medical Sciences, Tabriz, Iran; ^2^Nutrition Research Center, Department of Clinical Nutrition, Faculty of Nutrition and Food Sciences, Tabriz University of Medical Sciences, Tabriz, Iran; ^3^Research Center for Integrative Medicine in Aging, Aging Research Institute, Tabriz University of Medical Sciences, Tabriz, Iran; ^4^Endocrine Research Center, Tabriz University of Medical Sciences, Tabriz, Iran

**Keywords:** Fenugreek dry extract, Type 2 diabetes, Glycemic indices, Lipid profile, PAB

## Abstract

**Introduction:**

This study aims to determine the effects of fenugreek seed dry extract (FDE) on the glycemic indices, lipid profile, and prooxidant-antioxidant balance (PAB) in patients with type 2 diabetes (T2D).

**Methods:**

A double-blind randomized clinical trial was carried out on 54 individuals with T2D. Participants were randomly assigned to a FDE group (received 3 tablets containing 335 mg of FDE daily for 8 weeks) or a placebo group (received tablets containing microcrystalline cellulose). Anthropometric indices, physical activity, diet, fasting blood sugar (FBS), serum insulin, Homeostatic Model Assessment for Insulin Resistance (HOMA-IR), triglyceride (TG), total cholesterol (TC), low-density lipoprotein-cholesterol (LDL-C), high-density lipoprotein (HDL-C), and PAB were assessed.

**Results:**

An eight-week intake of 3 tablets containing 335 mg of FDE decreased serum insulin (*P*=0.016, *P*<0.001), HOMA-IR (*P*=0.009, *P*<0.001), TG (*P*<0.001, *P*=0.001), and PAB (*P*<0.001, *P*<0.001) compared to the baseline, in both placebo and intervention groups respectively. TC decreased significantly compared to the baseline in the placebo group (*P*=0.028), while HDL-C increased in the FDE group compared to the baseline (*P*<0.001) and placebo group (*P*=0.014).

**Conclusion:**

In the present study even though changes of parameters were more in intervention group compared to the control group, we did not observe any significant differences between studied groups except for HDL-C. However, the effects might become apparent with a higher dosage, longer study duration, or a larger sample size compared to the placebo group. Further clinical trials are needed in this regard.

## Introduction

 Type 2 diabetes (T2D) is a significant chronic metabolic condition globally ^1^, responsible for 90% of diabetes cases.^2^ This condition arises from a malfunction in insulin secretion, insulin resistance, or both.^3^ Unfortunately, the prevalence of this disease is increasing, with an estimated 642 million people expected to be affected by 2040.^4^ Several factors contribute to the rising prevalence of T2D, including genetic predisposition, obesity and overweight, aging population, socioeconomic status, social and environmental influences, and reduced physical activity as urbanization continues. ^5^

 Individuals with T2D often experience dyslipidemia due to the impact of insulin deficiency and insulin resistance on lipid metabolism enzymes and pathways.^6^ Additionally, the prevalence of overweight and obesity among individuals with T2D is a contributing factor to the increased levels of blood lipids in this population. ^7^ Also, chronic elevation of blood sugar levels can lead to increased oxidative stress and complications in the blood vessels of diabetic patients. ^8^ Diabetes mellitus is one of the main causes of cardiovascular diseases (CVD), blindness, kidney failure, and amputation of lower limbs. ^9^ Lifestyle changes including weight loss, being physically active, a healthy diet, and regular use of medications are the first line of treatments in diabetes control. ^10,11^ However, due to the side effects caused by medications, the use of herbal drugs has been given renewed attention today. Fenugreek (Trigonella foenum-graecum.Linn.) is a natural plant that is utilized for managing complications and addressing a range of illnesses, due to its high content of flavonoids, alkaloids, saponins, and other antioxidants. It has been documented to possess medicinal properties including anti-diabetic, anti-atherosclerosis, anti-inflammatory, and anti-cancer effects. ^12,13^ Studies on animals have used alcoholic or aqueous extracts of fenugreek seeds to explore the potential advantages of fenugreek in managing and preventing diabetes risk factors such as body composition, blood sugar levels, lipid profile, oxidative stress, and inflammation. ^14-17^ Evidence indicates that fenugreek is more potent than other plant compounds with beneficial properties in lowering fasting blood sugar (FBS) and insulin resistance (HOMA-IR). ^18^ Moreover, it has been shown that this substance has enhanced the lipid profile in individuals with T2D. ^19^ Therefore, fenugreek may be regarded as a viable treatment option for T2D. Studies on fenugreek seed extraction have shown that the amount of seeds consumed is significantly higher than that of extract obtained (up to 5000 times). Furthermore, all the beneficial compounds in the seeds are retained in much higher concentrations in the extract compared to the seeds. As a result, the adequate and effective dosage of fenugreek extract may be significantly lower. ^20^ The present study aimed to investigate the effect of fenugreek seed dry extract (FDE) on primary outcomes including glycemic indices, lipid profile, and prooxidant-antioxidant balance (PAB), and secondary outcomes such as physical activity, weight changes, and dietary intakes in people with T2D.

## Materials and Methods

###  Study population

**Figure 1:**
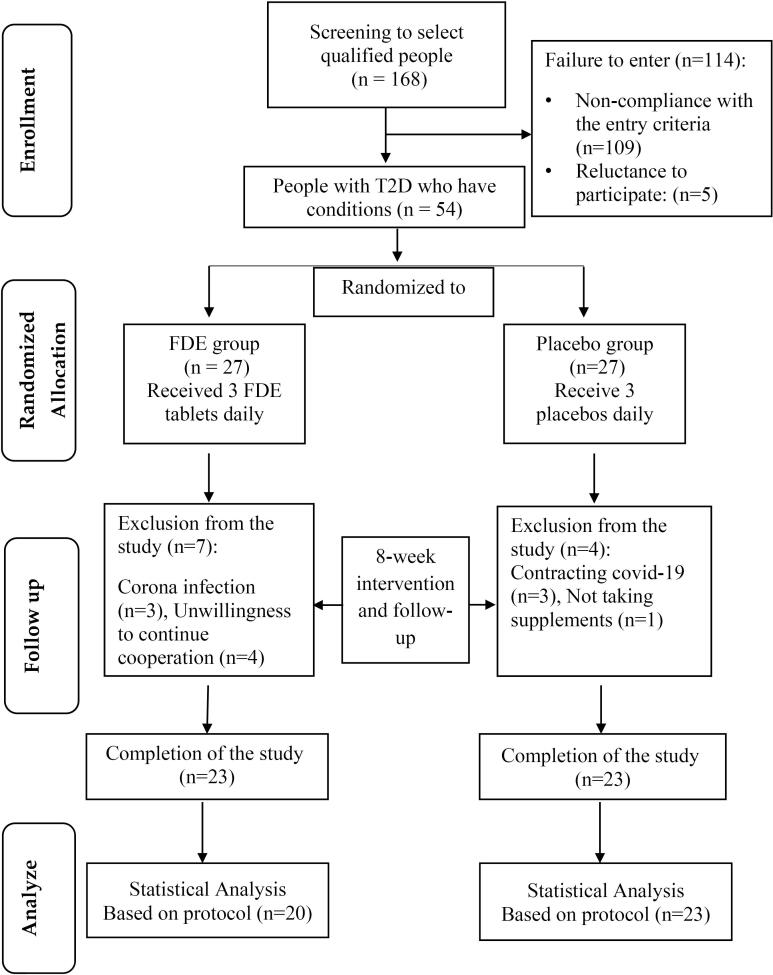
Flowchart of study implementation steps. FDE: fenugreek dry extract, T2D: Type 2 diabetes

 This randomized, parallel intervention study conformed to the ethical guidelines of the 1975 Declaration of Helsinki. It was approved by the Ethics Committee of Tabriz University of Medical Sciences (Ethical Code: IR-TBZMED.REC.1400.242 and registration code of Iran clinical trials: IRCT20210407050881N1). This double-blind (participants and investigator) randomized controlled clinical trial study was conducted on patients with T2D who were referred to the Tabriz Diabetes Association and their disease was confirmed and diagnosed by an endocrinologist. The sample size was determined based on the primary information obtained from the study by Hadi et al ^13^ for the FBS variable. For α value equal to 0.05 (confidence level of 95%) and a power of 90%, the sample size was computed using this formula:


$$n=\frac{{\left(z_{1}-\frac{\alpha }{2}+z_{1}-\beta \right)}^{2}(\delta_{1}^{2}+\delta_{2}^{2})}{{\left(\mu_{1}-\mu_{2}\right)}^{2}},$$

(α = 0.05 and β = 0.1) as 20 subjects per group. Considering the loss of 35%, 27 patients with T2DM (35-67 y) were determined for each group, therefore 54 individuals diagnosed with T2D, aged between 30 and 65 years, and with a minimum of 6 months of T2D history, were chosen based on meeting specific criteria. Written informed consent was obtained from patients and then randomly allocated into the intervention group (n = 27) or the control group (n = 27). The study objectives and methods were presented to the participants, and their written consent was obtained. Using the random block method with a block size of 4, participants were assigned to either the FDE or placebo groups in a 1:1 allocation ratio, based on gender and BMI matching, with the assistance of Random allocation software (RAS) (Figure 1). The eligibility criteria for this study involve individuals aged 30-65 years with a minimum of six months of diabetes history (by the American Diabetes Association criteria), a body mass index (BMI) between 25 and 35 kg/m2, use of blood sugar-lowering medications, no recent use of herbal remedies for at least three months before the study, and willingness to participate in the study. Individuals were excluded from the study if they were pregnant or breastfeeding, had kidney, liver, digestive, thyroid, or rheumatism conditions, had allergies to plants in the Fabaceae family, or were smokers. The analysis did not include participants who discontinued further participation in the study, consumed less than 90% of FDE, and used insulin injections.

###  Intervention

 Participants in the intervention group were given 3 tablets of 335 mg Fenugreek seed dry extract and 46.4 micrograms of Luteolin daily, and those in the control group received 3 tablets of 46.4 micrograms of Luteolin per tablet and microcrystalline cellulose for 8 weeks. The FDE and placebos were manufactured by Barij Essence Company and were designed to be identical in shape, size, and color to maintain the double-blind nature of the study. To perform concealment in a double-blind clinical trial, the allocation code was given to each of the intervention and control groups by a third party who was not from the study investigators and remained hidden from the researchers. Also, supplements were allocated according to the sequence of patients’ referrals, and all researchers and patients were unaware of the treatment codes by the end of the study. Regular phone calls were conducted with the patients every 10 days to monitor their intake of FDE and address any potential issues. The contents of the bottles given to each participant were reviewed to evaluate their adherence to the study.

###  Evaluation of anthropometric indices, physical activity, and dietary intake

 Body weight was measured using a digital scale (Seca, Hamburg, Germany) with an accuracy of 0.1 kg, and height was measured using a stadiometer (Seca) with 0.5 cm accuracy. BMI was calculated as weight in kilograms divided by height in meters squared and was assessed pre-and post-intervention. The participants’ level of physical activity was assessed using the short version of the International Physical Activity Questionnaire (IPAQ).^21^ Participants’ dietary intake was recorded using a 24-hour dietary recall for 3 days, including 2 weekdays and 1 weekend day, and analyzed with the Nutritionist IV program. All participants were asked to maintain their usual diet and physical activity during the study.

###  Measurement of biochemical indices

 At the beginning and end of the study, 10 cc blood samples were taken from all patients after 10 to 12 hours of fasting at baseline and post-intervention. Biochemical indices including FBS were measured using enzymatic and colorimetric methods (Pars Azmoun kit (Pars Azmoun, Iran)), The enzyme-linked immunosorbent assay (ELISA) method and the Monobind human insulin kit were used to measure serum insulin levels. Insulin resistance was determined using the HOMA-IR index ^22^,


$$HOMA-IR=\frac{Fasting\;blood\;glucose\left[\frac{mmol}{L}\right]\times Fasting\;insulin\left[\frac{mU}{L}\right]}{22.5}.$$

The enzymatic method was used to measure the serum concentration of triglyceride, total cholesterol, and HDL cholesterol, using a Pars Azmoun kit (Pars Azmoun, Iran). LDL-c was determined using the Freidewald formula^23^: LDL-c (mg/dl) = TC-HDL-TG/5. The measurement of serum pro-oxidant-antioxidant balance (PAB) was measured by a method utilizing tetramethylbenzidine (TMB) - TMB cation to determine the redox state, specifically the pro-oxidant-antioxidant balance. Various studies have investigated and confirmed the diagnostic significance of this marker. ^24^

###  Statistical analysis

 The statistical software IBM SPSS Statistics (IBM SPSS Statistics, IBM, Armonk, NY, USA) version 26 was used for data analysis. The normality of data distribution was evaluated using the Kolmogorov-Smirnov (KS) test. Quantitative data were reported as mean ± standard deviation. Qualitative data were presented as frequency (percentage). A paired samples t-test was employed to compare pre-and post-intervention normal variables within each group. Independent samples t-test was utilized to compare changes between groups. To assess the overall impact of the intervention adjustments were made for confounding factors such as sex, age, physical activity, drug used, energy and CHO intake, and baseline values using the statistical method of analysis of covariance (ANCOVA). Per protocol method applied for data analysis. *P* < 0.05 was considered statistically significant.

## Results

###  Study population characteristic

 A total of 43 diabetic patients were included in the final examination, with 20 (10 males and 10 female) individuals in the FDE group and 23 (11 males and 12 female) individuals in the placebo group. Throughout the study, 3 individuals in the placebo group and 3 in the FDE group contracted COVID-19. Additionally, 4 subjects in the FDE group and 1 subject in the placebo group were excluded from the study for personal reasons. Table 1 presents the general characteristics of the subjects under study. The average age of patients in the FDE group was 58.80 ± 6.23 years, while in the placebo group, it was 55.83 ± 8.12 years, with no significant difference between the two groups. Additionally, there were no significant differences between the groups in terms of sex (*P* = 0.887), marital status (*P* = 0.295), education level (*P* = 0.205), job status (*P* = 0.555), and type of drugs consumed (*P* = 0.484).

**Table 1:** General characteristics of participants in the two studied groups

**Variables**	**Placebo (n=23)**	**FDE (n=20)**	***P *** **value**
**Age** (year; mean ± SD)	55.83 (8.12)	58.80 (6.23)	0.192^a^
**Sex**			0.887^a^
Male	11 (47.8)	10 (50)	
Female	12 (52.2)	10 (50)	
**BMI (mg/kg**^2^**)**			
Baseline (mean ± SD)	29.32 ± 2.93	29.59 ± 4.44	0.818^b^
End of study (mean ± SD)	28.93 ± 2.90	29.66 ± 4.29	0.512^b^
Change (mean ± SD)	-0.38 ± 0.84	0.07 ± 0.7	0.061^b^
*P* value	0.039^c^	0.642^c^	
**Education**			0.205^b^
Less than diploma	11 (47.80	15 (75)	
Diploma	6 (26.1)	3 (15)	
Academic	6 (26.1)	2 (10)	
**Job**			0.555^a^
Unemployed	15 (65.2)	14 (73.7)	
Employed	8 (34.8)	5 (26.3)	
**Marital**			0.295^a^
Single	4 (17.4)	7 (35)	
Married	19 (82.6)	13 (65)	
**Physical activity level**			0.275^a^
Low	13 (56.5)	8 (40)	
Medium	7 (30.4)	5 (25)	
Intense	3 (13)	7 (35)	
**Drugs History**			0.484^d^
Metformin + sulfonylurea	6 (26.1)	8 (40)	
Metformin	5 (21.7)	5 (25)	
Sulfonylurea	1 (4.3)	2 (10)	
Metformin + sulfonylurea DPP4 + SGL2	11 (47.8)	5 (25)	

Values are reported as mean (± SD) for age and body mass index (BMI) and frequency (percentage) for other data. FDE, fenugreek dry extract; DPP4, dipeptidyl peptidase 4; SGL2, sodium-glucose cotransporter-2. ^a’b^Independent samples t-test. ^c^Paired Samples T-test. ^d^Fisher’s exact test.

###  Dietary intake of study subjects

Table 2 displays the results of energy and macronutrient intake in the two groups at the beginning and end of the study. There were no significant differences between the groups for energy and macronutrient intake before and after intervention. However, at the end of the study, the placebo group showed a considerable decrease in energy (*P* = 0.009) and carbohydrate intake (*P* < 0.001) compared to the baseline, while protein and fat intake remained unchanged. Conversely, the FDE group did not show significant changes in energy and macronutrient intake compared to the baseline.

**Table 2:** Comparison of energy intake and macronutrients in the two study groups before and after the intervention

**Variables**	**Placebo (n=23)**	**FDE (n=20)**	***P*** ** value**
Energy (Kcal/day)			
Before	2095.17 (365.13)	2065.45 (392.01)	0.798^b^
After	1900.73 (273.83)	1986 (378.19)	0.264, 0.134^d^
Mean changes (95% CI), P^a^	-194.43 (-335.91,-52.95),0.009	-79.45 (-255.64,96.74), 0.357	
Protein (g/day)			
Before	73.74 (20.52)	77.59 (19.76)	0.536^b^
After	66.30 (16.84)	67.20 (17.7)	0.936^c^,0.877^d^
Mean changes (95% CI), P^a^	-7.44 (-16.88, 2/00), 0.117	-10.39 (-23.27, 2.47), 0.107	
Carbohydrate (g/day)			
Before	320.11 (56.13)	320.68 (75.56)	0.977^b^
After	276.20 (50.91)	311.59 (84.25)	0.05^c^, 0.147^d^
Mean changes (95% CI), P^a^	-43.9 (-66.62, -21.18), 0.001	-9.09 (-42.67, 24.47), 0.577	
Fat (g/day)			
Before	56.67 (23.03)	53.36 (21.93)	0.634^b^
After	60.29 (15.20)	53.39 (25.45)	0.324^c^
Mean changes (95% CI), P^a^	3.62 (-6.04, 13.29), 0.445	0.03 (-6.64, 6.71), 0.991	

Values are reported as mean (± SD). FDE, fenugreek dry extract. ^a^ Paired samples t-test. ^b^ independent samples t-test. ^c^ Analysis of covariance (ANCOVA) with adjustment of baseline values (model 1).

###  Physical activity of study subjects

 According to the results shown in Table 3, there was no significant difference in the average level of physical activity between the two study groups at baseline and post-intervention. In addition, there were no significant changes within groups at the end of the study (*P* = 0.275).

**Table 3:** Comparison of physical activity values in the two study groups before and after the intervention

**Variables**	**Placebo (n=23)**	**FDE (n=20)**	***P *** **value**
Physical activity (METs)			
Before	643 (364.5, 1874.25)	719.75 (259.87, 2879.5)	0.559^b^
After	990 (396,2772)	1044 (445.5, 2229.75)	0.785^b^, 0.462^c^
Mean changes (95% CI), P^a^	0.13 (-0.15,-0.42),0.333	-0.02 (-0.20,0.16), 0.812	

Values are reported as median (25 and 75). FDE, fenugreek dry extract. ^a^ Paired samples t-test. ^b^ independent samples t-test. ^c^ Analysis of covariance (ANCOVA) with adjustment of baseline values (model 1).

###  The Effect of FDE on glycemic indices

Table 4 displays the changes in glycemic indices of the patients. The findings indicated no statistically significant differences in FBS, serum insulin, and HOMA-IR levels between the groups at the beginning and end of the study (P ≥ 0.05). Results remained unchanged after adjusting for baseline and confounding factors including age, sex, drug use, changes in energy intake, carbohydrate (CHO) intake, and changes in physical activity. However, within-group comparison using paired t-test revealed a notable decrease in serum insulin and HOMA-IR levels in both the FDE (*P* < 0.001 and *P* < 0.001) and placebo groups (*P* = 0.016 and *P* = 0.009) respectively.

**Table 4:** Comparison of glycemic index values in participants in two study groups

**Variables**	**Placebo (n=23)**	**FDE (n=20)**	***P *** **value**
FBS (mg/dL)			
Before	153.47 (64.57)	173.4 (77.32)	0.406^b^
After	143.69 (54.48)	152.7 (54.42)	0.563^b^,0.896^c^, 0.531^d^
Mean changes (95% CI), P^a^	-9.78 (-9.67,29.24),0.320	-20.70 (-0.47,41.87), 0.083	
Serum insulin (µIU/ml)			
Before	3.78 (1.78)	3.59 (1.10)	0.679^b^
After	2.88 (1.29)	2.50 (0.07)	0.335^b^,0.385^c^, 0.295^d^
Mean changes (95% CI), P^a^	-0.89 (-1.45, -0.34), 0.016	-1.08 (-1.60, -0.56), < 0.001	
HOMA-IR			
Before	1.31 (0.53)	1.48 (0.69)	0.356^b^
After	0.99 (0.49)	0.94 (0.44)	0.793^b^,0.434^c^, 0.265^d^
Mean changes (95% CI), P^a^	-0.31 (-0.54, -0.08), 0.009	-0.54 (-0.82, -0.25), < 0.001	

Values are reported as mean (± SD). FDE, fenugreek dry extract. ^a^ Paired samples t-test. ^b^ independent samples t-test. ^c^ Analysis of covariance (ANCOVA) with adjustment of baseline values (model 1). ^d^ analysis of covariance (ANCOVA) by adjusting baseline values, age, sex, drug used, changes in energy intake, CHO intake, and changes in physical activity (model 2).

###  The Effect of FDE on lipid profile

 According to the data in Table 5, there were no significant differences in the average TC, TG, and LDL-C indices between the two study groups at baseline and post-intervention. For HDL-C there was no significant difference between groups at baseline, while the results of analysis of covariance (ANCOVA) by adjusting on baseline values, age, sex, drug used, changes in energy intake, CHO intake, and changes in physical activity showed a significant difference between groups at the end of the study (*P* = 0.014). Further analysis within the placebo group revealed a substantial change in total cholesterol (*P* = 0.028) and triglycerides (*P* < 0.001), while a significant decrease in triglycerides and HDL levels (*P* < 0.001) was observed within the FDE group.

**Table 5:** Comparison of lipid profile in participants in two study groups

**Variables**	**Placebo (n=23)**	**FDE (n=20)**	***P*** ** value**
Total Cholesterol (mg/dL)			
Before	163.08 (33.90)	147.8 (32.22)	0.139^b^
After	146.04 (31.96)	145.7 (26.12)	0.970^b^,0.562^c^, 0.139^d^
Mean changes (95% CI), P^a^	-17.04 (-32.07,-2.01),0.028	-2.10 (-18.13,13.93), 0.787	
Triglyceride (mg/dL)			
Before	170.69 (71.11)	166.65 (63.63)	0.940^b^
After	111.21 (13.76)	116 (26.12)	0.244^b^, 0.166^c^,0.093^d^
Mean changes (95% CI), P^a^	-59.47 (-88.01, -30.93), < 0.001	-50.65 (-78.22, -23.07), < 0.001	
LDL-c (mg/dL)			
Before	88.64 (32.85)	75.94 (33.25)	0.187^b^
After	81.766 (36.47)	76.42 (27.21)	0.579^b^, 0.961^c^, 0.126^d^
Mean changes (95% CI), P^a^	-6.87 (-21.11, 7.36), 0.327	0.48 (-16.54, 17.51), 0.953	
HDL-c (mg/dL)			
Before	40.30 (5.41)	38.52 (5.88)	0.308^b^
After	42.03 (6.48)	46.07 (5.1)	0.030^b^, 0.013^c^, 0.014^d^
Mean changes (95% CI), P^a^	1.72 (-1.39, 4.84), 0.263	7.54 (4.56, 10.52), < 0.001	

Values are reported as mean (± SD). FDE, fenugreek dry extract. ^a^ Paired samples t-test. ^b^ independent samples t-test. ^c^ Analysis of covariance (ANCOVA) with adjustment of baseline values (model 1). ^d^ analysis of covariance (ANCOVA) by adjusting baseline values, age, sex, drug use, changes in energy intake, CHO intake, and changes in physical activity (model 2).

###  The Effect of FDE on PAB

Table 6 presents the findings regarding changes in the PAB index at baseline and after 8 weeks of intervention across two groups. Initially, there was no statistically significant difference in the average PAB index between the studied groups (*P* > 0.068). However, the FDE and placebo groups showed a notable decrease in PAB levels through intra-group comparison using a paired t-test (*P* < 0.001). Although the amount of reduction was greater in the intervention group compared to the placebo group, there was no significant difference in the mean of PAB between groups at the end of the study (*P* = 0.055). Adjusting on baseline values (*P* = 0.217) and the abovementioned confounding factors did not change the results (*P* = 0.736).

**Table 6:** Comparison of PAB in participants in two study groups

**Variables**	**Placebo (n=23)**	**FDE (n=20)**	***P*** ** value**
PAB			
Before	98.09 (20.26)	121.76 (33.68)	0.068^b^
After	64.02 (14.88)	74.52 (20.33)	0.055^b^,0.217^c^, 0.736^d^
Mean changes (95% CI), P^a^	-34.06 (-43.78,-24.35), < 0.001	-47.23 (-63.70,-30.77), < 0.001	

Values are reported as mean (± SD). FDE, fenugreek dry extract. ^a^ Paired samples t-test. ^b^ independent samples t-test. ^c^ Analysis of covariance (ANCOVA) with adjustment of baseline values (model 1). ^d^ analysis of covariance (ANCOVA) by adjusting baseline values, age, sex, drug used, changes in energy intake, CHO intake, and changes in physical activity (model 2).

## Discussion

 The purpose of the present double-blind randomized controlled clinical trial was to examine the impact of fenugreek seed extract on glycemic parameters, lipid profile, prooxidants, and antioxidant balance in individuals with T2D. The findings indicated that 8 weeks’ intake of three tablets containing 335 mg fenugreek seed extract helped decrease insulin levels and HOMA-IR in the intervention group compared to the baseline. However, there was no significant difference between groups at the end of the study. Although fenugreek seed extract reduced the FBS levels in the intervention group compared to the baseline and control group, this reduction was not statistically significant. It appears that increasing the intervention dose, extending the duration, or enlarging the study’s sample size could lead to substantial effects.

 The findings of the present study were aligned with some previous clinical trials. In these studies, 4 to 12 weeks of intervention with 1 gram to 15 grams of various types of fenugreek including seed, seed powder, seed extract, boiled, defatted, and germinated seed have shown no significant effects on FBS levels. ^25-32^ However, our findings were in contrast with the results of Kumar et al ^33^ who reported a significant decrease in FBS by 8 weeks intake of 5gram/day fenugreek seed powder in 50 patients with type 2 diabetes and also Gholaman et al study ^34^ in which a significant decrease in serum FBS but no significant changes in insulin levels and HOMA-IR was reported by 8 weeks consumption of 15 grams fenugreek seed. The difference in the observed results may be due to the differences in the specific characteristics of the Gholaman et al study’s participants (obese women with T2D), higher baseline level of FBS, higher insulin resistance due to obesity, potential difference between lifestyle, physical activity, and diet, and higher dose used, compared to our study. No significant changes in insulin levels were also reported in some other studies that had investigated the effects of 11 days, 6 weeks, and 8 weeks of about 1 gram of fenugreek seed extract consumption in healthy subjects ^27^, a woman with polycystic ovarian syndrome^26^ and healthy overweight male ^28^ respectively. In Kiss et al ^27^ and Bashtian et al ^26^ studies contrary to our results no significant change was also observed in the HOMA-IR. These differences may be attributed to the specific population studied, normal levels of the abovementioned parameters at baseline, and the methodological differences between studies.

 Based on research, the anti-diabetic effects of fenugreek can be attributed to 4-hydroxyisoleucine (4-HIL), which can increase insulin sensitivity and reduce insulin resistance, thereby improving glucose transport into liver cells through the phosphorylation of insulin receptors.^35^ In addition, fenugreek seeds contain high amounts of fiber, which can reduce the rate of glucose absorption after a meal. This may be a secondary mechanism for its hypoglycemic effect. ^36^

 Fenugreek has also been shown to have anti-hyperlipidemic effects. ^37-39^ The findings of the current study indicated that an 8-week intake of three tablets containing 335 mg of fenugreek seed extract led to a significant reduction in post-intervention TG and a notable increase in HDL-C compared to the baseline and the placebo group. However, there was no significant difference between groups for TG, TC and LDL-C at the end of the study. It seems that by increasing the intervention’s dose, the study duration, or the sample size, its effects can be significant.

 The results of the present study were in line with the findings of several clinical trials which reported increased levels of HDL-C ^33,34,40,41^ and decreased levels of TG^29,30,33,34,40-42^ by consuming 25 mg to 60 grams of different forms of fenugreek seed for 4 to 12 weeks. However, some clinical trials have shown no significant effects of fenugreek on HDL-C and TG levels. ^25,27-31,34,42-48^ In the Kumar et al^33^, Geberemeskal et al ^41^, Yousefi et al ^31^, and Verma et al ^49^ clinical trials, in contrast to the results of our study, a significant reduction in the levels of TC and LDL-C was observed compared to the baseline and also control group. These discrepancies may be due to the specific population studied (healthy, diabetic, and pre-diabetic subjects), normal or near normal levels of the abovementioned parameters at baseline, and the methodological differences including study design, sample size, intervention dose and type, duration and statistical methods used.

 Several hypotheses have been proposed regarding the anti-hyperlipidemic mechanism of fenugreek seeds. The decrease in TC, TG, and LDL-C levels, along with the increase in HDL-C levels, observed with fenugreek seed powder may be attributed to the crude fiber content in the seeds. On the other hand, in addition to its high fiber (total fiber content 48%), fenugreek also has a significant biological level of saponins that have hypocholesterolemic effects. Diosgenin is a furostanol saponin that prevents the absorption of cholesterol and thus reduces the level of cholesterol in the liver and increases the excretion of cholesterol in the bile, and finally the concentration of serum cholesterol decreases. Furthermore, 4-Hydroxyisoleucine can increase HDL-C levels and lower total cholesterol and triglycerides. ^50^

 In addition to the anti-diabetic and anti-hyperlipidemic effects of fenugreek, its antioxidant properties have also been investigated in several studies. In most studies stress oxidative imbalance is assessed by measuring the amount of pro-oxidant and antioxidant separately which is costly. However, in the present study, we measured the PAB factor, which simultaneously provides an integrated measurement of pro-oxidant and antioxidant activity using a simple, fast, and cheap method. ^24^ The condition in which pro-oxidants outnumber antioxidant defenses is called PAB. This abnormality condition is defined as stress oxidative, which is followed by accretion in the presence of reactive oxygen species (ROS) in tissues. ^51^ The findings of the present study showed a significant decrease in the PAB in both studied groups compared to the baseline. The reduction rate in the intervention group was higher than in the placebo group. However, there was no significant difference between groups at the end of the study. By increasing the intervention dose or duration, significant changes may be made.

 To the best of our knowledge, there has been no study on the effects of fenugreek on PAB. However, conflicting results have been reported by clinical studies investigating the effects of fenugreek on routine stress oxidative markers. A significant increase in TAC and a significant decrease in MDA serum levels were observed in the Foroumandi et al study that supplemented 5 cc of fenugreek seed extract for 4 months in elderly people with mild to moderate Alzheimer’s disease. ^52^ In another study conducted by Tavakoly et al on 48 patients with type 2 diabetes, an intake of 15 grams of fenugreek seed powder for 8 weeks caused a significant increase in SOD levels, while there was no change in the serum levels of GPX and TAC. ^53^ These differences between studies may be due to the specific population studied, and the methodological differences including study design, sample size, intervention dose and type, duration, and statistical methods used.

 Based on evidence antioxidant effects of fenugreek are attributed to the presence of compounds such as polysaccharides (galactomannan), saponins (diosgenin), alkaloids (trigonelin), polyphenols, flavonoids, and amino acids (4-Hydroxyisoleucine (4-HIL)). ^36,54^ Trigonelline and 4-HIL are believed to exert their anti-inflammatory effects by modulating nuclear factor kappa (NF-κB) and influencing messenger RNA (mRNA) associated with inflammation and oxidative stress regulation. ^55,56^

 The current study has several strengths, including the matching of patients by gender and BMI, consideration of potential confounding variables, regular in-person and telephone contact with patients to enhance motivation and follow-up, assessment of patients’ dietary and physical activity habits, and creation of individualized meal plans for each participant. Using of PAB factor, which simultaneously provides an integrated measurement of pro-oxidant and antioxidant activity using a simple, fast, and cheap method was another important strength of our study. Nevertheless, our study had its limitations. The spread of COVID-19 led to a significant drop in sample volume, and we also faced time and financial constraints. In addition, LDL-C was calculated by the Friedewald formula (FF), which is less validated compared to the directly measured LDL. Furthermore, the lack of a specific biomarker for measuring fenugreek meant that we had to rely solely on the self-reported supplement consumption by the patients to evaluate compliance in this study.

## Conclusion

 In the present study, there was a noteworthy change in insulin, insulin resistance, TG, and PAB levels in the intervention and control groups compared to the baseline. However, the changes were more in the intervention group than in the control group. The effects might become apparent with a higher dosage, longer study duration, or a larger sample size. In addition, HDL-C levels increased significantly in the FDE group compared to the baseline and placebo group while TC decreased significantly in the placebo group compared to the baseline. Further investigation is required to confirm these findings.

## Acknowledgments

 The authors thank to Vice Chancellor of Tabriz University of Medical Sciences for their support and thank all the patients for their participation in this study. This study is the result of the Master of Science thesis for Fatemeh Chehregosha.

## Competing Interests

 The authors declared no potential conflict of interest.

## Ethical Approval

 The present study was approved by the Research Ethics Committees of Tabriz University of Medical Sciences. Ethical Code: IR-TBZMED.REC.1400.242.
